# Development of NP-Based Universal Vaccine for Influenza A Viruses

**DOI:** 10.3390/vaccines12020157

**Published:** 2024-02-02

**Authors:** Ekramy E. Sayedahmed, Nelly O. Elshafie, Andrea P. dos Santos, Chinnaswamy Jagannath, Suryaprakash Sambhara, Suresh K. Mittal

**Affiliations:** 1 Department of Comparative Pathobiology, Purdue Institute for Immunology, Inflammation and Infectious Disease, Purdue University Center for Cancer Research, College of Veterinary Medicine, Purdue University, West Lafayette, IN 47907, USA; esayedah@purdue.edu (E.E.S.); nelshafi@purdue.edu (N.O.E.); santos1@purdue.edu (A.P.d.S.); 2 Department of Pathology and Genomic Medicine, Center for Infectious Diseases and Translational Medicine, Houston Methodist Research Institute, Weill-Cornell Medicine, Houston, TX 77030, USA; cjagannath@houstonmethodist.org; 3 Influenza Division, Centers for Disease Control and Prevention, Atlanta, GA 30329, USA

**Keywords:** adenoviral vector, influenza vaccine, universal influenza vaccine, nucleoprotein, autophagy, autophagy-inducing peptide

## Abstract

The nucleoprotein (NP) is a vital target for the heterosubtypic immunity of CD8^+^ cytotoxic T lymphocytes (CTLs) due to its conservation among influenza virus subtypes. To further enhance the T cell immunity of NP, autophagy-inducing peptide C5 (AIP-C5) from the CFP10 protein of *Mycobacterium tuberculosis* was used. Mice were immunized intranasally (i.n.) with human adenoviral vectors, HAd-C5-NP(H7N9) or HAd-NP(H7N9), expressing NP of an H7N9 influenza virus with or without the AIP-C5, respectively. Both vaccines developed similar levels of NP-specific systemic and mucosal antibody titers; however, there was a significantly higher number of NP-specific CD8 T cells secreting interferon-gamma (IFN-γ) in the HAd-C5-NP(H7N9) group than in the HAd-NP(H7N9) group. The HAd-C5-NP(H7N9) vaccine provided better protection following the challenge with A/Puerto Rico/8/1934(H1N1), A/Hong Kong/1/68(H3N2), A/chukkar/MN/14951-7/1998(H5N2), A/goose/Nebraska/17097/2011(H7N9), or A/Hong Kong/1073/1999(H9N2) influenza viruses compared to the HAd-NP(H7N9) group. The autophagy transcriptomic gene analysis of the HAd-C5-NP(H7N9) group revealed the upregulation of some genes involved in the positive regulation of the autophagy process. The results support further exploring the use of NP and AIP-C5 for developing a universal influenza vaccine for pandemic preparedness.

## 1. Introduction

Influenza viruses still constitute a significant risk to human health worldwide. Approximately one billion human infections, three to five million severe cases, and about 300,000 to 500,000 deaths occur every year despite the availability of seasonal influenza vaccines [1,2]. Influenza viruses accumulate continuous antigenic changes due to immune pressure and faulty genome replication systems. This antigenic drift lowers the efficacy of seasonal influenza vaccination.

In addition to seasonal influenza viruses (H1N1, H3N2, and influenza B), both low or highly pathogenic avian influenza (HPAI) viruses like H5, H7, and H9 types can infect humans, thereby demonstrating their pandemic potential [3,4,5,6]. Since its appearance in Asia over two decades ago, the HPAI H5N1 viruses have spread to more than 60 countries on four continents and are still present in poultry in southern Asia and Africa [7]. In addition, H9N2 avian influenza virus can infect humans, and the avian influenza viruses (AIVs) of H7N2, H7N3, and H7N7 subtypes still lead to periodic infections [8,9,10]. In early 2010’s, a novel AIV H7N9 subtype appeared in China, responsible for over 1568 human infections leading to 616 deaths [11]. The evolution of AIV is still ongoing, and despite human-to-human transmission being rare, viral replication in the upper respiratory tract of humans can occur following the genetic recombination of avian influenza and human/swine influenza or mutations in one or more genes resulting in a novel pandemic influenza virus [12,13,14,15].

While candidate vaccines can be made for individual influenza strains, preparing significant vaccine stocks for each potential pandemic virus is impractical. Moreover, the nature of the pandemic influenza virus will only be known at the time of the pandemic. Therefore, it will be practical to develop a universal flu vaccine that could confer adequate protection against seasonal influenza A viruses (H1N1 and H3N2) as well as potential pandemic AIVs (H5N1, H7N7, H7N9, and H9N2).

The influenza virus’s internal protein, nucleoprotein (NP), is conserved in multiple subtypes and serves as a robust inducer of cytotoxic CD8 T lymphocyte (CTL) responses [16,17,18,19]. The non-neutralizing antibody responses help in viral clearance through antibody-dependent cell cytotoxicity (ADCC), complement-dependent lysis (CDL), or the induction of CD4 T helper cells [20,21]. Earlier, we demonstrated that the intramuscular (i.m.) vaccination of mice with the human adenoviral (HAd) type 5 (HAd5) vector expressing NP protein of an H5N1 virus was able to reduce the lung virus titers of H1, H3, H5, H7, and H9 influenza viruses by nearly 2.4, 1.9, 2.3, 2.4, and 1.4 logs, respectively [22]. Therefore, NP offers an excellent target for developing a universal influenza vaccine.

Originally, the autophagy process was recognized as a cell-survival cellular process through which catabolic digestion of unwanted cellular components is utilized to provide nutrients during stress. Interestingly, the autophagy process is involved in multiple cellular processes, including antigen presentation and immune responses [23,24,25,26]. Autophagy regulates both the MHC-II-dependent and MHC-I-dependent presentation of antigens in antigen-presenting cells (APCs) [27,28]. It thus serves a unique role in vaccine-induced immune responses in both mice and humans [29]. An autophagy-inducing peptide (AIP) C5 (AIP-C5) is a 22-amino-acid-long peptide from the secreted CFP10 protein of *Mycobacterium tuberculosis* (Mtb). This peptide significantly enhanced the cell-mediated immune (CMI) responses against an epitope from 85B protein and resulted in protection against Mtb challenge in mice when delivered through an Ad vector [30]. Here, we investigated whether the inclusion of the C5-AIP with the H7N9 NP gene significantly enhances T cell immune responses and broadens the protective efficacy of an Ad vector-based universal influenza vaccine candidate. Our results elucidated that intranasal (i.n.) immunization of mice with HAd vector expressing NP (H7N9) or C5-NP(H7N9) conferred complete protection against H1N1, H3N2, H5N2, H7N9, and H9N2 influenza viruses, signifying the importance of the route of immunization (i.n.), delivery vector (Ad), influenza antigen (NP), and the autophagy-inducing peptide (AIP-C5) in developing a universal influenza vaccine. In addition, there was no AIP-C5-mediated inflammatory response in the lungs, but it resulted in the upregulation of the genes involved in the autophagy pathway.

## 2. Results

### 2.1. Generation of HAd-NP(H7N9) and HAd-C5-NP(H7N9) Vectors

**Figure 1:**
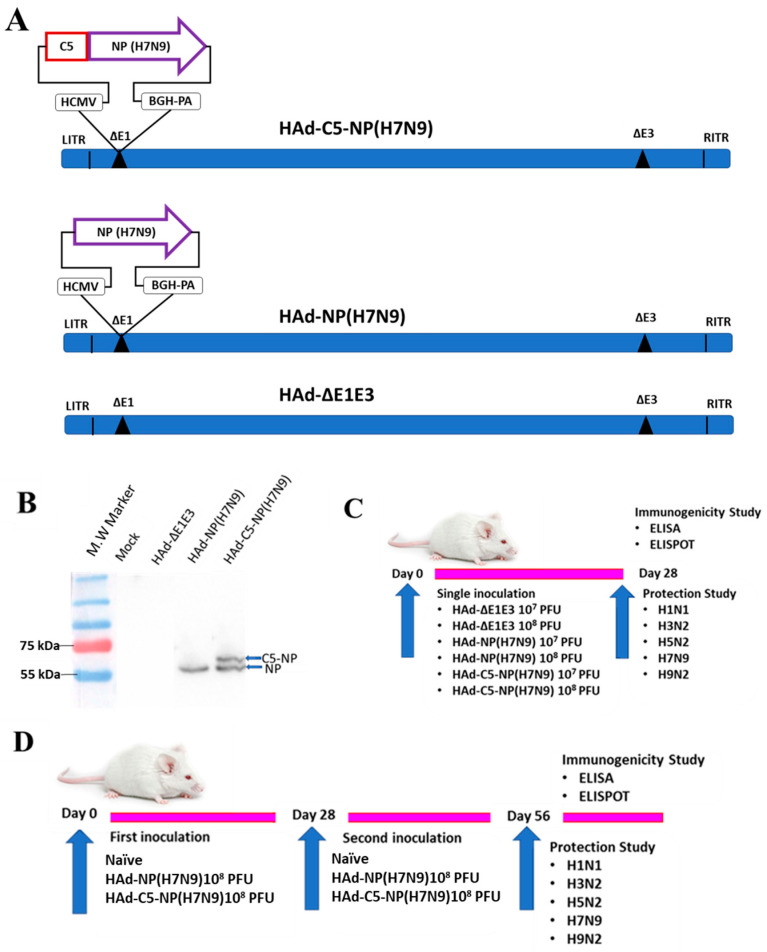
(**A**) Diagrammatic image of HAd−C5−NP(H7N9), HAd−NP(H7N9), and HAd-ΔE1E3 genomic representation. The cytomegalovirus (CMV) promoter and the bovine growth hormone (BGH) polyadenylation signal are flanking the NP(H7N9) or C5-NP(H7N9) genes. The drawings are not to scale. LITR, left inverted terminal repeat; RITR, right inverted terminal repeat; ΔE1, deletion of E1 region; ΔE3, deletion of E3 region; C5, C5-AIP; NP, nucleoprotein. (**B**) Immunoblot confirming expression of NP(H7N9) or C5-NP(H7N9) in HAd-NP(H7N9)- or HAd-C5-NP(H7N9)-infected 293 cells, respectively. Mock or HAd-ΔE1E3 infected cell extracts were used as a negative control. The molecular weight marker is shown on the left. Outlines of the one-dose (**C**) or the two-dose (**D**) animal inoculation study.

The HAd vectors [HAd-NP(H7N9) and HAd-C5-NP(H7N9)] containing the H7N9 NP gene of the A/Shanghai/02/2013(H7N9) influenza A virus without or with AIP-C5, respectively, were generated (Figure 1A) by the Cre recombinase-mediated recombination [31]. The gene cassette with the inserted NP genes was identified by vector DNA restriction analysis and sequencing of the PCR-amplified gene cassette. To confirm the expression of NP or C5-NP in 293 cells infected with HAd-NP(H7N9) or HAd-C5-NP(H7N9), the vector-infected cell extracts were processed for Western blot assay. Mock-infected cell extracts were used as a control. The presence of an approximately 56 kDa band in HAd-NP(H7N9)-infected cell extract or two bands of about 56 and 61 kDa in HAd-C5-NP(H7N9)-infected cell extract suggests the expression of NP or C5-NP, respectively (Figure 1B). Two bands in the HAd-C5-NP(H7N9)-infected cell extract represent C5-NP and cleaved NP due to the presence of P2A cleavage peptide between AIP-C5 and NP.

### 2.2. Development of Similar Levels of Humoral Immune Responses in Single Dose Immunized Mice i.n. with HAd-NP(H7N9) or HAd-C5-NP(H7N9)

The overall experimental design for the one-dose regimen is outlined (Figure 1C). The mice were inoculated i.n. with 1 × 10^7^ or 1 × 10^8^ plaque-forming units (PFU) of HAd-NP(H7N9), HAd-C5-NP(H7N9), or HAd-ΔE1E3.

**Figure 2:**
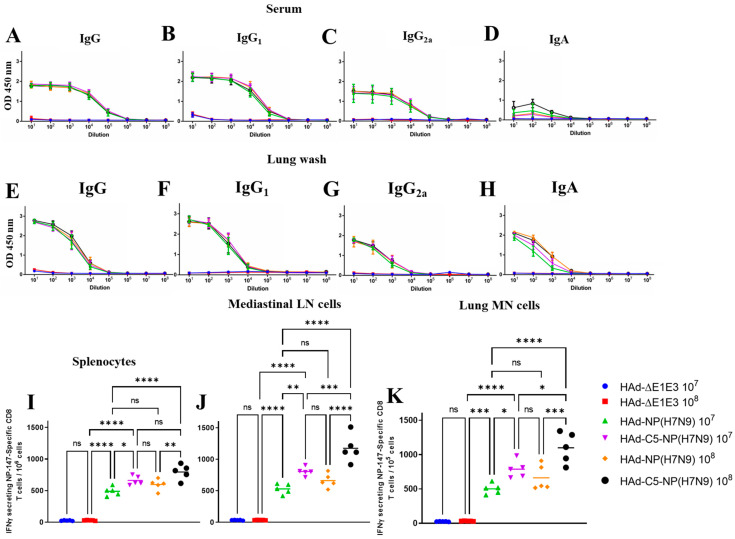
Immunogenicity of HAd−NP(H7N9) or HAd−C5−NP(H7N9) in inoculated mice. BALB/c mice six to eight weeks old (5 animals/group) were vaccinated once intranasally (i.n.), as described in the material and methods section. After vaccination for four weeks, blood samples were collected and used to monitor the development of NP-specific IgG (**A**), IgG_1_ (**B**), IgG_2a_ (**C**), and IgA (**D**) antibody responses by ELISA. Lung washes were also collected to monitor the development of mucosal NP-specific IgG (**E**), IgG_1_ (**F**), IgG_2a_ (**G**), and IgA (**H**) antibody responses by ELISA. The data are displayed as the mean ± standard deviation (SD) of the optical density (OD). Enhancement in the number of NP-specific IFN-γ-secreting CD8 T cells following immunization with HAd-C5-NP(H7N9) was monitored at 4 weeks post-vaccination in the spleen (**I**), mediastinal lymph node (LN) (**J**), and lung mononuclear (MN) Cells (**K**) by enumerating NP-specific IFN-γ-secreting CD8 T cells by ELISpot using the NP-147 peptide. ns, non-significant at *p* > 0.05; *, significant at *p* < 0.05; **, significant at *p* < 0.01; ***, significant at *p* < 0.001; and ****, significant at *p* < 0.0001.

As expected, there were no detectable hemagglutination inhibition (HI) or virus-neutralizing (VN) antibody titers against an H7N9 influenza virus. NP-specific antibodies are not considered virus-neutralizing; however, NP-specific ADCC and CDL have been observed [32]. Low levels of NP-specific IgA, but very high levels of NP-specific IgG, IgG_1,_ and IgG_2a_, were found in mice sera immunized either with HAd-NP(H7N9) or HAd-C5-NP(H7N9) (Figure 2A–D). Both the HAd-NP(H7N9) and HAd-C5-NP(H7N9) groups showed similar levels of humoral immune responses in the serum samples, indicating that the inclusion of AIP-C5 did not have a significant impact on the levels of systemic humoral immune responses. The control groups inoculated i.n. with HAd-∆E1E3 did not induce anti-NP humoral immune response levels above the background (Figure 2A–D). No significant dose-dependent differences in systemic humoral immune responses were observed in vaccinated animals.

Furthermore, the development of NP-specific humoral immune responses at the mucosal level was also determined. High levels of NP-specific IgA, IgG, IgG_1,_ and IgG_2a_ were observed in the lung washes of mice inoculated with HAd-NP(H7N9) or HAd-C5-NP(H7N9) (Figure 2E–H). Both the HAd-NP(H7N9) and HAd-C5-NP(H7N9) groups showed similar levels of humoral immune responses in the lung washes. The lung washes collected from HAd-∆E1E3 i.n. inoculated groups did not elicit anti-NP humoral immune responses above the background (Figure 2E–H). Again, there were no noticeable dose-dependent differences in NP-specific mucosal humoral immune responses in the vaccinated groups.

### 2.3. Enhancement of NP-Specific CD8 T Cell Responses by HAd-C5-NP(H7N9) Compared to HAd-NP(H7N9)

The influenza virus internal protein NP is conserved across multiple subtypes and is a robust inducer of CTLs and non-neutralizing antibody responses [32]. NP-specific CD8 T cell responses are vital for the virus clearance after infection and perform a critical role in homologous and heterosubtypic influenza virus protection [33,34]. In addition, AIP-C5 has been shown to enhance CMI responses due to antigen processing through autophagy [30].

To investigate the effect of AIP-C5 on CD8 T cell responses in the HAd-C5-NP(H7N9) group compared to the HAd-NP(H7N9) group, splenocytes, mediastinal lymph node (LN) cells, and lung mononuclear (MN) cells were collected to monitor the development of CD8 T cell responses. There was a significant increase in NP-specific IFN-γ-secreting CD8 T cells in the spleen (Figure 2I), mediastinal LN (Figure 2J), and lung MN cells (Figure 2K) in the HAd-C5-NP(H7N9) group than in the HAd-NP(H7N9) group, suggesting that the AIP-C5 led to the enhancement of CD8 T cell responses. Also, there were dose-dependent increases in the CMI responses in vaccinated groups (Figure 2I–K).

### 2.4. Protection of Mice Immunized Once with HAd-C5-NP(H7N9) or HAd-NP(H7N9) following Challenge with H1N1, H3N2, H5N2, H7N9 and H9N2 Influenza A Viruses

To determine homo- and heterosubtypic protection, HAd-C5-NP(H7N9), or HAd-NP(H7N9), the immunized mice were challenged i.n. with two lethal doses of 50 (LD_50_) of A/Puerto Rico/8/1934(H1N1), 5 LD_50_ of A/Hong Kong/1/68(H3N2), and 100 mouse infectious dose 50 (MID_50_) of A/chukkar/MN/14951-7/1998(H5N2), A/goose/Nebraska/17097/2011(H7N9), or A/Hong Kong/1073/1999(H9N2). Since A/Puerto Rico/8/1934(H1N1) or A/Hong Kong/1/68(H3N2) influenza virus causes morbidity or mortality in mice, the vaccine efficacy was evaluated by monitoring the morbidity or mortality in mice for two weeks following the challenge. Whereas A/chukkar/MN/14951-7/1998(H5N2), A/goose/Nebraska/17097/2011(H7N9), or A/Hong Kong/1073/1999(H9N2) did not induce symptoms in mice, the reduction in lung viral titers after the challenge in vaccinated animals was used to evaluate the vaccine protection efficacy.

**Figure 3:**
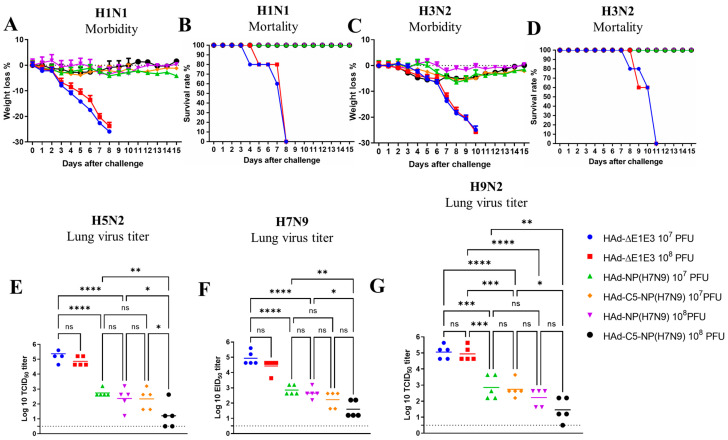
Protection efficacy of single i.n. vaccination of mice with Had-NP(H7N9) or Had-C5-NP(H7N9). At 4 weeks post-inoculation, immunized animal groups were challenged with 2 lethal doses of 50 (LD_50_) of A/Puerto Rico/8/1934(H1N1) (**A**,**B**) or 5 LD_50_ of A/Hong Kong/1/68(H3N2) (**C**,**D**). (**A**,**C**) Morbidity and (**B**,**D**) mortality after challenge were monitored. (**E**–**G**). Groups were challenged with 100 mouse infectious dose 50 (MID_50_) of A/chukkar/MN/14951-7/1998(H5N2) (**E**), A/goose/Nebraska/17097/2011(H7N9) (**F**), or A/Hong Kong/1073/1999(H9N2) (**G**) influenza virus, and at 3 days post-challenge, the lungs were collected for virus titers. The data are shown as mean Log10 tissue culture infectious dose 50 (TCID_50_) or egg infectious dose 50 (EID_50_), and the detection limit was 0.5 Log10 TCID_50_ or EID_50_ per ml. ns, non-significant at *p* > 0.05; *, significant at *p* < 0.05; **, significant at *p* < 0.01; ***, significant at *p* < 0.001; and ****, significant at *p* < 0.0001.

Both HAd-C5-NP(H7N9)- and HAd-NP(H7N9)-immunized mouse groups with 10^7^ or 10^8^ PFU vaccine doses were protected from significant morbidity or mortality following the challenge with A/Puerto Rico/8/1934(H1N1) [Figure 3A,B] or A/Hong Kong/1/68(H3N2) [Figure 3C,D]. However, HAd-C5-NP(H7N9) immunized groups either with 10^7^ or 10^8^ PFU provided better protection following challenge with A/chukkar/MN/14951-7/1998(H5N2) [Figure 3E], A/goose/Nebraska/17097/201 (H7N9) [Figure 3F], or A/Hong Kong/1073/1999(H9N2) [Figure 3G] compared to the HAd-NP(H7N9) vaccinated groups. These results provided evidence that the AIP-C5-dependent augmentation of NP-specific CMI response confers better heterosubtypic protection.

### 2.5. Efficacy of a Two-Dose Regimen of HAd-C5-NP(H7N9) or HAd-NP(H7N9) in Eliciting Heterosubtypic Protection

**Figure 4:**
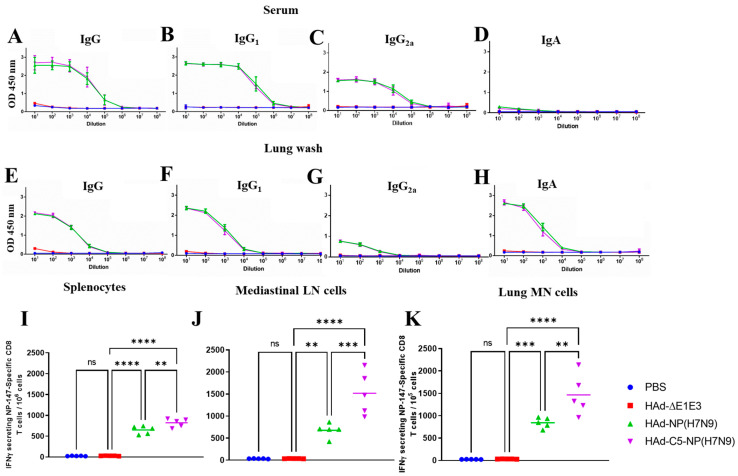
Immunogenicity of Had-NP(H7N9) or Had-C5-NP(H7N9) two-dose regimen inoculation. BALB/c mice six to eight weeks old (5 animals/group) were immunized twice intranasally (i.n.), as mentioned in the material and methods section. Three weeks post-boost, blood samples were collected and used to monitor the development of NP-specific IgG (**A**), IgG_1_ (**B**), IgG_2a_ (**C**), and IgA (**D**) antibody responses by ELISA. Three weeks post-boost, lung washes were also collected to monitor the development of mucosal NP-specific IgG (**E**), IgG_1_ (**F**), IgG_2a_ (**G**), and IgA (**H**) antibody responses by ELISA. ELISA results are the optical density (OD) readings as mean ± standard deviation (SD). Enhancement in the number of NP-specific IFN-γ-secreting CD8 T cells following immunization with HAd-C5-NP(H7N9) was monitored at 3 weeks post-booster in the spleen (**I**), mediastinal lymph node (LN) (**J**), and lung mononuclear (MN) cells (**K**) by enumerating NP-specific IFN-γ-secreting CD8 T cells by ELISpot using the NP-147 peptide. ns, non-significant at *p* > 0.05; **, significant at *p* < 0.01; ***, significant at *p* < 0.001; and ****, significant at *p* < 0.0001.

To determine whether the protection efficacy of HAd-C5-NP(H7N9) or HAd-NP(H7N9) could be further improved against H5N2, H7N9, and H9N2, the immunogenicity and challenge studies were repeated with a two-dose regimen of i.n. immunization by 1 × 10^8^ PFU HAd-C5-NP(H7N9) or HAd-NP(H7N9) (Figure 1D). Similar levels of NP-specific IgG, IgG_1_, IgG_2a,_ and IgA antibodies in serum samples (Figure 4A–D) or lung washes (Figure 4E–H) were observed in groups immunized with HAd-C5-NP(H7N9) or HAd-NP(H7N9). As expected, there was a statistically significant NP-specific CD8 T cells secreting IFN-γ in spleen (Figure 4I), mediastinal LN (Figure 4J), and lung MN cells (Figure 4K) in HAd-C5-NP(H7N9) inoculated mice than in HAd-NP(H7N9) group, revealing the importance of AIP-C5 in eliciting enhanced CD8 T cell responses.

**Figure 5:**
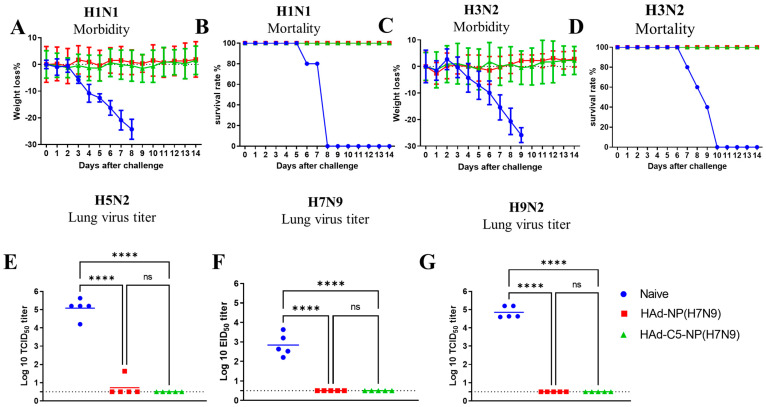
Protection of Had-NP(H7N9) or Had-C5-NP(H7N9) two-dose regimen. At 3 weeks post-booster, immunized animal groups were challenged with 2 lethal doses of 50 (LD_50_) of A/Puerto Rico/8/1934(H1N1) (**A**,**B**) or 5 LD50 of A/Hong Kong/1/68(H3N2) (**C**,**D**). (**A**,**C**) Morbidity and (**B**,**D**) mortality after challenge were monitored. (**E**–**G**). Groups were challenged with 100 mouse infectious dose 50 (MID_50_) of A/chukkar/MN/14951-7/1998(H5N2) (**E**), A/goose/Nebraska/17097/2011(H7N9) (**F**), or A/Hong Kong/1073/1999(H9N2) (**G**) influenza virus, and the lungs were collected 3 days post-challenge and lung viral titers were determined. The data are shown as mean Log10 tissue culture infectious dose 50 (TCID_50_) or egg infectious dose 50 (EID_50_), and the detection limit was 0.5 Log10 TCID_50_ or EID_50_ per ml. ns, non-significant at *p* > 0.05; and ****, significant at *p* < 0.0001.

Both HAd-C5-NP(H7N9)- and HAd-NP(H7N9)-immunized mouse groups were completely protected from the challenge with no morbidity or mortality from A/Puerto Rico/8/1934(H1N1) [Figure 5A,B] or A/Hong Kong/1/68(H3N2) [Figure 5C,D] influenza virus. In addition, both HAd-C5-NP(H7N9) and HAd-NP(H7N9) vaccinated mouse groups had complete protection after the challenge with A/chukkar/MN/14951-7/1998(H5N2) [Figure 5E], A/goose/Nebraska/17097/2011(H7N9) [Figure 5F], or A/Hong Kong/1073/1999(H9N2) [Figure 5G] influenza virus, except for one animal that showed a detectable lung virus titer in the HAd-NP(H7N9)-immunized group challenged with the H5N2 virus. Overall, the two-dose regimen suggests that enhanced heterosubtypic protection can be achieved with an NP-based mucosal vaccine.

### 2.6. Lung Histopathology of Mice Immunized i.n. with HAd-NP(H7N9) or HAd-C5-NP(H7N9)

**Figure 6:**
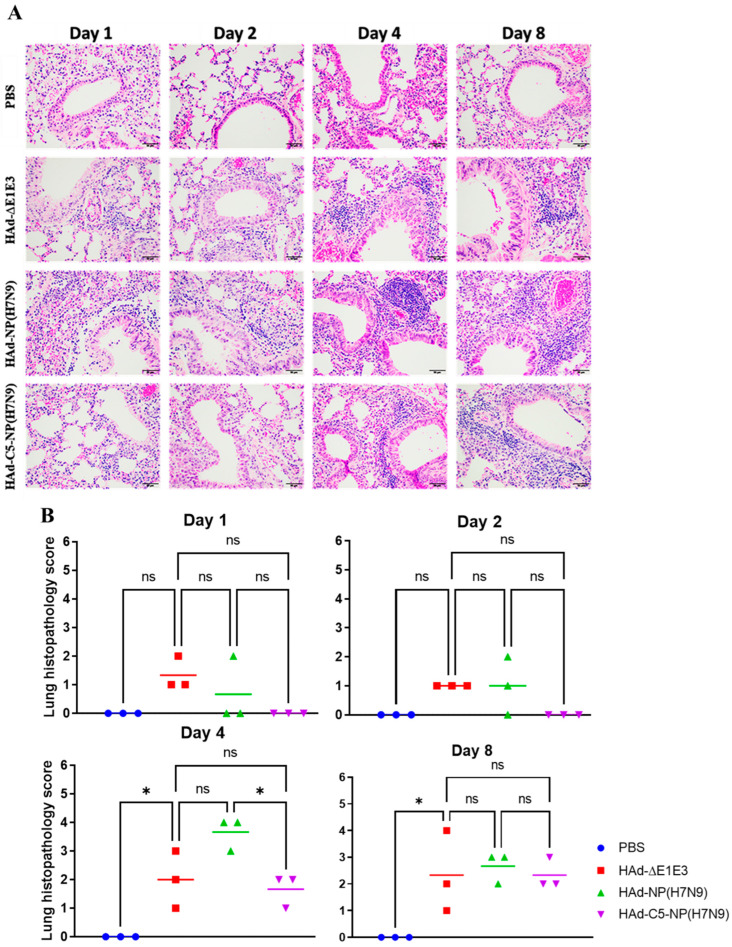
Histopathology of HAd−NP(H7N9)- or HAd−C5−NP(H7N9)-immunized mice lung tissues. (**A**) BALB/c mice (3 animals/group) were mock-immunized (PBS) or immunized intranasally (i.n.) with 10^8^ plaque-forming units (PFU) of HAd-∆E1E3, HAd-NP(H7N9), or HAd-C5-NP(H7N9). Animals were euthanized at 0.25-, 0.5-, 1-, 2-, 4-, and 8-day post-immunization, and the lung tissue samples were collected and processed for histopathology. Representative pictures of each group on days 1, 2, 4, and 8 post-immunization are shown (H&E, 200X). (**B**) The lung tissue histopathological scores from HAd-NP(H7N9)- or HAd-C5-NP(H7N9)-immunized mice. Lung tissue sections were blindly analyzed by a board-certified veterinary pathologist. ns, non-significant at *p* > 0.05; and *, significant at *p* < 0.05.

Since autophagy is a natural mechanism of removing cellular debris to improve cell functioning, it is anticipated that the addition of AIP-C5 together with NP should not impact inflammatory responses. To address this issue, mouse groups were mock-inoculated or immunized with HAd-∆E1E3, HAd-NP(H7N9), or HAd-C5-NP(H7N9), and at 0.25-, 0.5-, 1-, 2-, 4-, and 8-day post-inoculation, the animals were euthanized, and the lung samples were collected and processed for histopathology. No noticeable changes were observed at day 0.25 or 0.5 post-inoculation in any groups. In HAd-∆E1E3, HAd-NP(H7N9), and HAd-C5-NP(H7N9) groups, there were increases in peribronchiolar lymphocytes peaking at day 4 (Figure 6A). Histopathology scores for the HAd-C5-NP(H7N9) group were similar or lower compared to the HAd-NP(H7N9) or HAd-∆E1E3 group, suggesting that adding AIP-C5 to NP did not lead to enhanced inflammatory responses (Figure 6B).

### 2.7. Upregulation of the Genes Involved in the Autophagy Pathway by HAd-C5-NP(H7N9)

**Figure 7:**
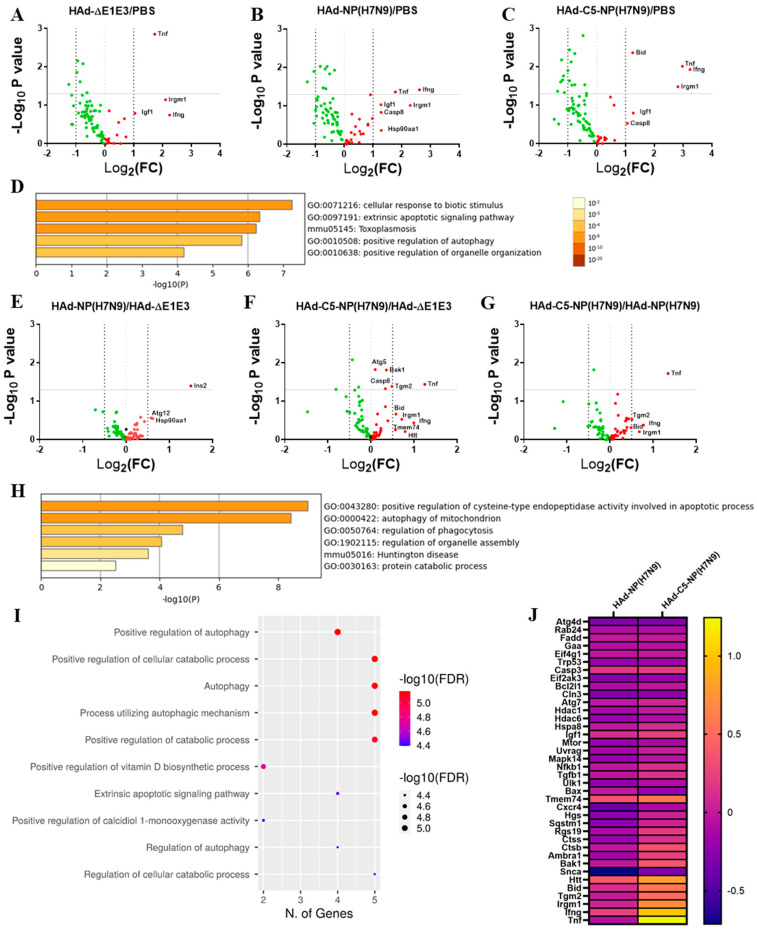
AIP-C5-mediated upregulation of genes involved in autophagy. BALB/c mice (3 animals/group) were mock-inoculated with PBS or inoculated i.n. once with 1 × 10^8^ PFU of Had-NP(H7N9), Had-C5-NP(H7N9), or HAd-ΔE1E3. The animals were euthanized at 24 h post-inoculation, and the lungs were collected for total RNA extraction. The RT^2^ mouse autophagy PCR array was used to analyze the differentially expressed genes in all study groups. (**A**) The differentially expressed genes between the HAd-∆E1E3 and the PBS groups are presented as a volcano plot. (**B**) The differentially expressed genes between the HAd-NP(H7N9) and the PBS groups are presented as a volcano plot. (**C**) The differentially expressed genes between the HAd-C5-NP(H7N9) and the PBS groups are presented as a volcano plot. (**D**) A bar graph showing the biological pathways of the significantly upregulated gene list input of the HAd-C5-NP(H7N9) group over the PBS group, colored by the *p*-values, is presented using the Metascape web analysis tool. (**E**) The differentially expressed genes between the HAd-NP(H7N9) and the HAd-∆E1E3 groups are presented as a volcano plot. (**F**) The differentially expressed genes between the HAd-C5-NP(H7N9) and the HAd-∆E1E3 groups are presented as a volcano plot. (**G**) The differentially expressed genes between the HAd-C5-NP(H7N9) and the HAd-NP(H7N9) groups are presented as a volcano plot. (**H**) A bar graph showing the biological pathways of the significantly upregulated gene list input of the HAd-C5-NP(H7N9) group over the HAd-∆E1E3 group, colored by the *p*-values, is presented using the Metascape web analysis tool. (**I**) A dot blot chart of the gene ontology enrichment analysis for the biological pathways involved in the upregulated gene list of the HAd-C5-NP(H7N9) group using ShinyGO v0.76 is shown. (**J**) A heat map of the differentially expressed genes of the HAd-NP(H7N9) group and HAd-C5-NP(H7N9) group compared to the HAd-∆E1E3 group.

To evaluate whether the AIP-C5-mediated upregulation of the genes involved in the autophagy occurs, mock-infected or HAd-∆E1E3-, HAd-NP(H7N9)-, or HAd-C5-NP(H7N9)-infected mice were euthanized at 24 h post-infection, and the lung samples were collected for RNA extraction. RNA samples were used for an autophagy PCR array to analyze the differentially expressed genes by the parametric unpaired t-test compared to the PBS group (Figure S1A). The inoculation with the empty vector (HAd-∆E1E3) downregulated most of the genes involved in the autophagy process (Figure 7A); however, significant upregulation of the tumor necrosis factor (Tnf) gene with 1.7 log_2_ fold change (FC) was observed (Figure 7A). In addition to Tnf (1.77 log_2_ FC), there was significant upregulation of the interferon-gamma (Ifng) gene (2.6 log_2_ FC) in HAd-NP(H7N9)-inoculated animals (Figure 7B). In the HAd-C5-NP(H7N9)-inoculated group, significantly higher expression of the BH3 interacting domain death agonist (Bid) gene (1.25 log_2_ FC) and the immunity-related GTPase family M member 1 (Irgm1) gene (2.8 log_2_ FC), in addition to the Tnf (2.97 log_2_ FC) and Ifng (3.2 log_2_ FC) genes (Figure 7C). These differentially expressed genes were uploaded to Metascape [35] for functional enrichment analysis. The enrichment analysis provided enriched terms of gene ontology (GO) representing various biological processes and Kyoto Encyclopedia of Genes and Genomes (KEGG) pathways. The most enriched terms are demonstrated in Figure 7D,H. The most enriched terms involved in the biological process pathways are associated with positive apoptotic regulation, autophagy in the mitochondrion, and phagocytosis.

The differentially expressed genes of the HAd-NP(H7N9) or HAd-C5-NP(H7N9) group in comparison with the HAd-∆E1E3 group were also analyzed (Figure S1B). Compared with the HAd-ΔE1E3 group, the expression of the autophagy-related genes Ins2, Atg12, and Hsp90aa1 was increased in the HAd-NP(H7N9) group (Figure 7E). On the other hand, the HAd-C5-NP(H7N9) group demonstrated the upregulation of Atg 5, Bak1, Casp8, Tgm2, Tmem74, Bid, Irgm1, Htt, Ifng, and Tnf compared to the HAd-∆E1E3 group (Figure 7F). Compared to the HAd-NP(H7N9) group, there was a higher expression of Bid, Tgm2, Irgm1, Ifng, and Tnf in the HAd-C5-NP(H7N9) group (Figure 7G,J). The Metascape gene annotation enrichment analysis of the HAd-C5-NP(H7N9) group showed that the upregulated genes are involved in several biological processes, including autophagy and phagocytosis (Figure 7H). The ShyniGo web analysis tool gene ontology (GO) used for the annotation analysis of the HAd-C5-NP(H7N9) group’s upregulated genes revealed the importance of these genes in several biological processes and KEGG pathways, especially autophagy (Figure 7I). The HAd-C5-NP(H7N9) group’s upregulated genes are categorized by function and defined using high-level GO terms (Table S1). The Interactive and hierarchical clustering relationship between the enriched pathways of the HAd-C5-NP(H7N9) group’s upregulated genes indicates the involvement of the autophagy and apoptosis pathways (Figures S2 and S3).

## 3. Discussion

Ad vector-based vaccines have demonstrated excellent promise for developing effective vaccines against several pathogens, including the Ebola virus and severe acute respiratory syndrome coronavirus 2 (SARS-CoV-2) in preclinical [36,37,38,39,40] and clinical [41,42,43,44] studies, and were licensed under Emergency use authorization [45]. Moreover, Ad vector-based influenza vaccines expressing hemagglutinin (HA) [46,47,48], neuraminidase (NA) [49,50], NP [51,52], matrix protein 1 (M1) [53,54,55], or immunogenic domains or epitopes [56,57] have been developed, which showed great potential in providing significant protection against influenza viruses in experimental animals or human clinical trials. In addition, the issue of Ad vector immunity could be addressed by using less common HAds as well as nonhuman Ads vaccine platforms [58,59,60]. A nanoparticle-based vaccine carrying the four HAs of seasonal influenza viruses resulted in antibody responses similar to or higher than the quadrivalent influenza vaccines in animal models [61]. Immunized animals were protected from heterologous viruses due to the development of broadly protective antibody responses to the HA stem part. In a phase I trial, chimeric HA-based vaccines in healthy adults generated broad and durable cross-reactive antibodies against the HA stem domain [62].

NP is one of the conserved proteins of the influenza virus, and following infection, the NP-specific CD8 T cell responses play a critical role in recovery [63,64]. One of the objectives of this study was to investigate whether the mucosal expression of NP could provide complete protection against seasonal influenza A viruses (H1N1 and H3N2) and against potential pandemic AIVs (H5N2, H7N9, and H9N2). The other objective was to determine whether the inclusion of AIP-C5 with NP could impact the development of adaptive immune responses leading to improved protective efficiency against multiple influenza A viruses. The importance of NP as a potential target for designing broadly protective influenza vaccines has been investigated in several studies. Some studies have described broad but partial protection with Ad or other viral vector-based NP vaccines [65,66]. Other conserved influenza antigens like M1, HA2 (HA stalk domain), M1, and/or M2 ectodomain with NP in Ad vectors or other viral vectors have been utilized to broaden the vaccine protection efficacy [54,67,68]. These studies used the systemic route of inoculation to deliver viral vector-based vaccine formulations leading to variable protection efficacy.

In this study, we elucidated that i.n. vaccination of mice with HAd-NP(H7N9) or HAd-C5-NP(H7N9) elicited comparable humoral immune responses (IgG, IgG_1_, IgG_2a_, and IgA). However, the inclusion of AIP-C5 with NP led to significantly higher CMI responses as measured by the number of NP-specific IFN-γ-secreting CD8 T cells in the spleen, mediastinal LN, and lung MN cells. Moreover, the histopathological analysis showed that the addition of AIP-C5 to NP did not induce inflammation or other lung pathology. The only observed change in the lungs was an accumulation of lymphocytes in the peribronchiolar areas, which corroborates with an increase in the T cell response. Autophagy regulates both MHC-II-dependent [69] and MHC-I-dependent [70] presentation of antigens in APCs. It thus serves a unique role in immune responses induced by vaccination in both mice and humans [29]. A unique transcriptome of genes controlling autophagy, antigen processing, and lysosome traffic was observed in dendritic cells infected with a bovine Ad vector (BAdv85C5) expressing AIP-C5 with an Mtb epitope (Ag85B-p25) [30]. Moreover, dendritic cells or macrophages infected with BAdv85C5 led to enhanced antigen presentation to CD4 T cells, and this response was partly dependent on autophagy.

The whole lung RNA transcriptomic analysis of all the groups by RT^2^ Profiler PCR Array showed the ability of AIP-C5 to upregulate the expression of several genes (Tnf, Irmg1, Ifng, and Bid) involved in the autophagy pathway. The Tnf gene codes for a proinflammatory cytokine can initiate autophagy in several cell types, including epithelial cells [71]. The Irmg1 gene product regulates the functions of macrophages and T cells [72]. The Ifng gene product is essential in eliminating intracellular pathogens by inducing autophagy through a poorly understood mechanism [73]. The Bid gene product is a pro-apoptotic member of the Bcl-2 protein family and is involved in apoptosis and cell death through autophagy [74]. Also, the Bid protein plays a role in inflammation and innate immunity independently of its apoptotic function [75]. These upregulated genes indicate the role of AIP-C5 as a molecular adjuvant in positively regulating autophagy, antigen presentation, and immune responses.

The development of immunity in the respiratory tract plays a vital role in conferring complete protection against homologous and heterosubtypic influenza viruses. The developing higher CD8 T cell responses against NP due to AIP-C5 may lead to improved protection against influenza viruses. It has been reported that i.m. immunization of Ad vector expressing NP is responsible for roughly 2.4, 1.9, 2.3, 2.4, or 1.4 logs decline in lung virus titers of H1, H3, H5, H7, and H9 influenza viruses, respectively [22]. For this reason, we did not pursue the adoptive transfer of NP-specific CTLs and/or immune serum samples to determine the role of NP-specific CTLs and antibodies in heterosubtypic protection against influenza viruses. The genome-wide transcriptomic analyses of APCs, T, or natural killer (NK) cells in the lungs may help discover the factors critical for developing broadly protective immunity.

Our previous study has shown that the expression of green fluorescent protein (GFP) by different Ad vectors can be detected in the lungs for 16 days after the inoculation of mice by HAd-GFP vector [76]. This duration of expression is long enough to stimulate a good immune response against the transgene. Studies have shown that the immune responses with Ad vectored vaccines can last for a year or more in a mouse model [77]. Although the HAd-C5-NP(H7N9) and HAd-NP(H7N9) showed very promising results as broadly protective influenza vaccine candidates, the pre-existing immunity against HAds is widely prevalent [78] and can affect the vaccine efficacy, particularly following the repeat immunization with the same vector. To avoid this drawback, we are currently developing a broadly protective influenza vaccine using a bovine Ad vector which has shown its ability to avoid HAd pre-existing immunity [79].

It is imperative to develop an effective universal influenza vaccine for improved protection against seasonal influenza viruses and influenza pandemic preparedness. The results suggest that a mucosal Ad vector-based vaccine expressing NP with AIP-C5 could be the basis for developing a universal influenza vaccine. It will be essential to confirm the level and range of the protective efficacy of HAd-C5-NP(H7N9) in other influenza animal models such as ferrets. Further investigations are necessary to define the role of NP-specific ADCC in protection, the duration of protective immunity, and the memory of NP-specific B and T cell subsets.

## 4. Material and Methods

### 4.1. Cell Lines

HEK293 (human embryonic kidney cells expressing HAd5 E1 proteins; ATCC: CRL-1573TM) [80] and 293Cre (293 cells expressing Cre recombinase) were kindly gifted by professor Frank Graham, Department of Biology, McMaster University, Hamilton, Ontario, Canada [81]. BHH2C (bovine-human hybrid clone 2C) was a cell line developed in our lab [82], and MDCK.2 (Madin-Darby canine kidney; ATCC: CRL-2936TM) cell lines were grown as monolayer cultures in Corning™ Dulbecco’s Modification of Eagle’s Medium (DMEM) (Fisher Scientific, Waltham, MA, USA) containing 10% reconstituted fetal bovine serum (Hyclone, Logan, UT, USA) and gentamycin (50 µg/mL).

### 4.2. Ad Vectors and Influenza Viruses

The NP gene of the A/Shanghai/02/2013(H7N9) influenza virus (Accession#YP_009118476) without [NP(H7N9)] or with AIP-C5 [C5-NP(H7N9)] was synthesized commercially (GenScript, Piscataway, NJ, USA). The NP(H7N9) or C5-NP(H7N9) gene cassette containing the cytomegalovirus (CMV) promoter and bovine growth hormone (BGH) polyadenylation signal were cloned into the HAd E1 shuttle plasmid. The vectors [HAd-NP(H7N9) and HAd-C5-NP(H7N9)] were created following a Cre-recombinase-mediated site-specific recombination method as described [83]. The generation of HAd-ΔE1E3 (HAd-5 E1 and E3 deleted empty vector) has been described previously [83]. HAd-NP(H7N9), HAd-C5-NP(H7N9), and HAd-ΔE1E3 were grown in 293 cells and titrated in BBH2C cells as described earlier [84]. For immunization studies, the vectors were purified via cesium chloride density-gradient ultracentrifugation [83].

A/Puerto Rico/8/1934(H1N1), A/Hong Kong/1/68(H3N2), A/chukkar/MN/14951-7/1998(H5N2), A/goose/Nebraska/17097/2011(H7N9), and A/Hong Kong/1073/1999(H9N2) were grown in embryonated hen eggs and titrated in the eggs and/or MDCK.

The NP amino acid sequence similarities between all influenza strains used in this study and influenza B viruses are shown in Table S2.

### 4.3. Immunogenicity and Challenge Studies in Mice

Studies were conducted in 6- to 8-week-old BALB/c mice (Jackson Laboratory, Bar Harbor, ME, USA). The mice (30 animals/group) were inoculated i.n. twice (at 3 weeks interval) with PBS or 1 × 10^8^ PFU of HAd-NP(H7N9) or HAd-C5-NP(H7N9). For the single-dose regimen, animal groups were also vaccinated i.n. with 1 × 10^7^ PFU or 1 × 10^8^ PFU of HAd-NP(H7N9), HAd-C5-NP(H7N9), or HAd-ΔE1E3. Four weeks after inoculation (single-dose regimen) or three weeks post second inoculation (two-dose regimen), 5 animals/group were anesthetized using Ketamine/Xylazine (100 mg/Kg ketamine and 10 mg/Kg xylazine), the blood samples were collected through the retro-orbital bleeding, and the lung washes were prepared from one lung as described in [85]. The serum samples as well as the lung wash were used to evaluate the humoral immune responses. The second lung was used to separate the lung MN cells via lymphocyte separation medium (#25-072-CV, Corning, Corning, NY, USA, Thermo Fisher Scientific). The lung MN cells splenocytes and mediastinal LNs cells were used to evaluate CMI responses.

The remaining 25 animals per group (five animals with each virus) were challenged i.n. either with 2 LD_50_ of A/Puerto Rico/8/1934(H1N1), 5 LD_50_ of A/Hong Kong/1/68(H3N2), or 100 MID_50_ of A/chukkar/MN/14951-7/1998(H5N2), A/goose/Nebraska/17097/2011(H7N9), or A/Hong Kong/1073/1999(H9N2). For the lethal challenge, mice were checked daily for morbidity and mortality for two weeks after the challenge. Otherwise, the lungs were collected on day 3 post-challenge for the nonlethal challenge, and the lung viral titers were measured in MDCK or embryonated chicken eggs [22].

### 4.4. Enzyme-Linked Immunosorbent Assay (ELISA)

The ELISA was accomplished as previously described [86]. Ninety-six-well flat-bottom ELISA plates (Fisher Scientific, Waltham, MA, USA) were first incubated overnight at 4 °C with purified H7N9 NP protein (0.5 µg/mL) [MyBioSource, Inc., San Diego, CA, USA]. Following blocking with 2% bovine serum albumin (BSA), log10 diluted serum or lung wash samples were incubated for 2 h at room temperature. The plates are washed four times with PBS + tween. The horseradish peroxidase-conjugated anti-mouse IgG, IgG_1_, IgG_2a_, or IgA antibodies (Invitrogen, Fisher Scientific, Waltham, MA, USA) were added at the dilutions recommended by the manufacturer and incubated at room temperature for 2 h. A BD OptEIA™ ELISA set TMB substrate (BD Biosciences, La Jolla, CA, USA; Fisher Scientific) was used for color development. The reaction was stopped with a 2 N sulfuric acid solution. The SpectraMax^®^ i3x microplate reader (Molecular Devices, Sunnyvale, CA, USA) was used to monitor the optical density (OD) at 450 nm.

### 4.5. ELISpot Assay

The IFN-γ ELISpot assay was pursued according to the previously described protocol [87]. The splenocytes, mediastinal LN cells, and lung MN cells were incubated with the NP147 (TYQRTRALV) peptide (H-2K^d^-restricted CTL epitope for NP) for 48 h at 37 °C in a CO_2_ incubator [88]. Subsequently, the plate was processed for IFN-γ ELISpot assay. The spot-forming units (SFU) were monitored using the AID iSpot Advanced Imaging Device (Autoimmun Diagnostika GmbH, Strassberg, Germany).

### 4.6. Histopathology

BALB/c mice (3 animals/group) were mock-immunized (PBS) or immunized i.n. with 10^8^ PFU of HAd-∆E1E3, HAd-NP(H7N9), or HAd-C5-NP(H7N9), and at 0.25, 0.5, 1, 2, 4, and 8 days, the animals were euthanized, and the lung and trachea samples were collected. The tissue samples were processed for histopathology at the Histology Research Laboratory, Center for Comparative Translational Research, Purdue University College of Veterinary Medicine. The tissue section slides were examined and graded for histopathological lesions by a board-certified veterinary pathologist who was not involved with the study design. The lungs were evaluated for the presence of atelectasis, edema, hemorrhage, congestion, alveolar thickness, presence of lymphocytes, and the presence of polymorphonuclear cells multiplied by the percentage of the section affected (cellSens Dimension Imaging Software version 1.18, Olympus Corp., Center Valley, PA, USA). The possible scores consisted of absent (0), minimal (1), mild (2), moderate (3), marked (4), and severe (5).

### 4.7. Total Lung RNA Extraction

BALB/c mice (3 mice/group) were inoculated with PBS or inoculated i.n. with 1 × 10^8^ PFU of HAd-NP(H7N9), HAd-C5-NP(H7N9), or HAd-ΔE1E3, euthanized at 24 h post-inoculation, lung samples were collected in cryovials and stored in RNAlater (Invitrogen, Thermo Fisher Scientific Corp.) at −80 °C till use. Total RNA extraction was carried out from each sample using the Monarch Total RNA Miniprep Kit (New England Biolabs Inc., Ipswich, MA, USA). The amount of RNA in each sample was calculated by measuring OD using a microplate spectrophotometer (Agilent Technologies, Santa Clara, CA, USA).

### 4.8. First-Strand DNA Synthesis

The RT^2^ First Strand Kit (QIAGEN Inc., Valencia, CA, USA) was used for the cDNA synthesis. DNA contamination from each RNA sample was removed by incubating in GE buffer. Then, the reverse transcription mix was used to synthesize the first DNA strand using the following reaction conditions: 42 °C for 15 min followed by 95 °C for 5 min. The samples were kept at −20 °C till use for the real-time polymerase chain reaction (PCR).

### 4.9. Real-Time PCR Using Autophagy PCR Array

RT^2^ Profiler PCR Array Mouse Autophagy (QIAGEN Inc.) was used to study the expression profiles of 84 genes involved in autophagy. The cDNA from each sample was diluted and mixed with the RT^2^ SYBR Green ROX qPCR Mastermix (QIAGEN Inc.). Briefly, 25 µL from the cDNA-master mix was added to each well of the PCR array 96-well plate and loaded into the QuantStudio 3 Real-Time PCR System (Thermo Fisher Scientific). The thermal cycling conditions were 95 °C for 10 min (1 cycle) and 95 °C for 15 s, followed by 60 °C for 1 min for 40 cycles. The cycle threshold values for all wells were exported to an Excel spreadsheet, and the data analysis was conducted using the GeneGlobe Data Analysis Center (https://geneglobe.qiagen.com/us/analyze (accessed on 28 August 2022)). The GeneGlobe Data Analysis Center uses several web-based tools for data analysis. The Excel and GraphPad Prism 6 statistics were used to validate the data analysis results.

### 4.10. Function and Pathway Enrichment Analysis

ShinyGo (http://bioinformatics.sdstate.edu/go/ (accessed on 30 August 2022)) and Metascape (https://metascape.org (accessed on 30 August 2022)) bioinformatics data analysis tools were used to determine the differentially expressed genes and their functional pathways. The enrichment outcomes and functionalities of the differentially expressed genes are exhibited using ShinyGO, another data analysis tool [89]. The false discovery rate (FDR) was set at 0.05.

### 4.11. Statistical Analyses

At a *p*-value < 0.05, statistically significant differences were established. The Bonferroni post-test in one-way was applied to determine the statistical significance. The parametric unpaired *t*-test was used to determine the significance of the differentially expressed genes between two groups in RT^2^ Profiler PCR Arrays. The results are presented as log_2_ fold change (log_2_ FC) and −log_10_ *p*-value. The RT^2^ Profiler PCR Arrays results are displayed as volcano plots using GraphPad Prism 9.

## Data Availability

Data are contained within the article and Supplementary Materials.

## Supplementary Materials

The following supporting information can be downloaded at: https://www.mdpi.com/article/10.3390/vaccines12020157/s1, Table S1. HAd-C5-NP(H7N9)-mediated upregulated genes that are grouped by functional categories and defined using high-level GO terms. Table S2: Influenza viruses nucleoprotein Percent Identity Matrix (PIM) - created by Clustal2.1. Figure S1. Heat maps of the differentially expressed genes with fold change value for HAd-∆E1E3, HAd-NP(H7N9), or HAd-C5-NP(H7N9) group compared to the PBS group (A), and the differentially expressed genes with fold change value for HAd-NP(H7N9), or HAd-C5-NP(H7N9) group compared to the HAd-∆E1E3 group (B). Figure S2. Interactive plot showing the relationship between the enriched pathways of the up-regulated genes in the HAd-C5-NP(H7N9) group. Significantly enriched gene sets are displayed with darker nodes. The size of the node represents the relative number of gene sets. Thicker edges represent an increased number of overlapped genes. Figure S3. A hierarchical clustering tree summarizing the correlation among significant pathways of upregulated genes in the HAd-C5-NP(H7N9) group. Pathways with many shared genes are clustered together. The size of the dots indicates increased *p*-values.
