# Supplementary material for: Development of NP-Based Universal Vaccine for Influenza A Viruses
Source: Vaccines (Basel). 2024 Feb 2;12(2):157. doi: 10.3390/vaccines12020157 (PMC10892571; doi:10.3390/vaccines12020157)
Supplement: Supplementary file 1 [file vaccines-12-00157-s001.zip › vaccines-2834540-supplementary.pdf]

**Table S1.** HAd-C5-NP(H7N9)-mediated upregulated genes that are grouped by functional categories and defined using high-level GO terms.

| N | High-level GO category | Genes | N | High-level GO category | Genes |
|---|---|---|---|---|---|
| 6 | Catabolic process | Bid Tnf Htt Irgm1 Tmem74 Ifng | 2 | Response to abiotic stimulus | Tnf Htt |
| 5 | Autophagy | Bid Htt Irgm1 Tmem74 Ifng | 2 | Anatomical structure morphogenesis | Tnf Ifng |
| 5 | Regulation of signaling | Bid Tnf Htt Irgm1 Ifng | 2 | Regulation of cell adhesion | Tnf Ifng |
| 5 | Cellular component biogenesis | Bid Tnf Htt Irgm1 Ifng | 2 | Modulation of the process of other organisms | Irgm1 Ifng |
| 5 | Regulation of response to stimulus | Bid Tnf Htt Irgm1 Ifng | 2 | Regulation of locomotion | Tnf Ifng |
| 4 | Response to stress | Bid Tnf Irgm1 Ifng | 2 | Leukocyte activation | Tnf Ifng |
| 4 | Response to external stimulus | Tnf Htt Irgm1 Ifng | 2 | Cell motility | Tnf Ifng |
| 4 | Response to biotic stimulus | Bid Tnf Irgm1 Ifng | 2 | Regulation of the developmental process | Tnf Ifng |
| 4 | Regulation of cellular component biogenesis | Bid Tnf Htt Ifng | 2 | Leukocyte migration | Tnf Ifng |
| 4 | Establishment of localization | Bid Tnf Htt Ifng | 2 | Maintenance of location | Tnf Htt |
| 4 | Cellular localization | Bid Tnf Htt Ifng | 2 | Regulation of multicellular organismal process | Tnf Ifng |
| 4 | Regulation of biological quality | Bid Tnf Htt Ifng | 2 | Localization of cell | Tnf Ifng |
| 4 | Regulation of molecular function | Bid Tnf Htt Ifng | 2 | Regulation of action potential | Tnf Ifng |
| 3 | Immune system process | Tnf Irgm1 Ifng | 1 | Cell killing | Ifng |
| 3 | Regulation of immune system process | Tnf Irgm1 Ifng | 1 | Behavior | Htt |
| 3 | System process | Tnf Htt Ifng | 1 | Growth | Ifng |
| 3 | Immune response | Tnf Irgm1 Ifng | 1 | Leukocyte homeostasis | Ifng |
| 3 | Cell population proliferation | Bid Tnf Ifng | 1 | Somatic diversification of immune receptors | Ifng |
| 3 | Cell cycle process | Bid Tnf Htt | 1 | Activation of immune response | Ifng |
| 3 | Regulation of localization | Tnf Htt Ifng | 1 | Myeloid cell homeostasis | Ifng |
| 3 | Macromolecule localization | Bid Tnf Ifng | 1 | Response to endogenous stimulus | Tnf |
| 3 | Biological process involved in interspecies interaction between organisms | Tnf Irgm1 Ifng | 1 | Antigen processing and presentation | Ifng |
| 3 | Response to other organism | Tnf Irgm1 Ifng | 1 | Regulation of cell killing | Ifng |
| 2 | Biological adhesion | Tnf Ifng | 1 | Killing of cells of other organism | Ifng |
| 2 | Locomotion | Tnf Ifng | 1 | Regulation of growth | Ifng |
| 2 | Immune effector process | Tnf Ifng | 1 | Taxis | Ifng |
| 2 | Production of molecular mediator of immune response | Tnf Ifng | 1 | Anatomical structure formation involved in morphogenesis | Tnf |
| 2 | Immune system development | Tnf Ifng | 1 | Detection of stimulus | Tnf |
| 2 | Cell adhesion | Tnf Ifng | | | |

N, number of genes.

Table S2: Influenza viruses nucleoprotein Percent Identity Matrix (PIM) - created by Clustal2.1

| | | | | | | | | | |
|---|---|---|---|---|---|---|---|---|---|
| 1: | **A/Shanghai/02/2013_H7N9** | 100.00 | 92.57 | 91.57 | 97.39 | 97.59 | 96.99 | 37.60 | 37.80 |
| 2: | **A/PuertoRico/8/1934_H1N1** | 92.57 | 100.00 | 94.38 | 93.98 | 94.18 | 93.17 | 37.60 | 37.80 |
| 3: | **A/HongKong/01/1968_H3N2** | 91.57 | 94.38 | 100.00 | 92.57 | 92.77 | 91.77 | 37.80 | 38.01 |
| 4: | **A/Chukkar/MN/14951-7/1998_H5N2** | 97.39 | 93.98 | 92.57 | 100.00 | 99.40 | 97.19 | 37.80 | 38.01 |
| 5: | **A/goose/Nebraska/17097/2011_H7N9** | 97.59 | 94.18 | 92.77 | 99.40 | 100.00 | 97.19 | 37.80 | 38.01 |
| 6: | **A/Quail/HongKong/G1/97_H9N2** | 96.99 | 93.17 | 91.77 | 97.19 | 97.19 | 100.00 | 38.21 | 38.41 |
| 7: | **B/Florida/4/2006_Yamagata** | 37.60 | 37.60 | 37.80 | 37.80 | 37.80 | 38.21 | 100.00 | 99.46 |
| 8: | **B/Brisbane/60/2008_Victoria** | 37.80 | 37.80 | 38.01 | 38.01 | 38.01 | 38.41 | 99.46 | 100.00 |

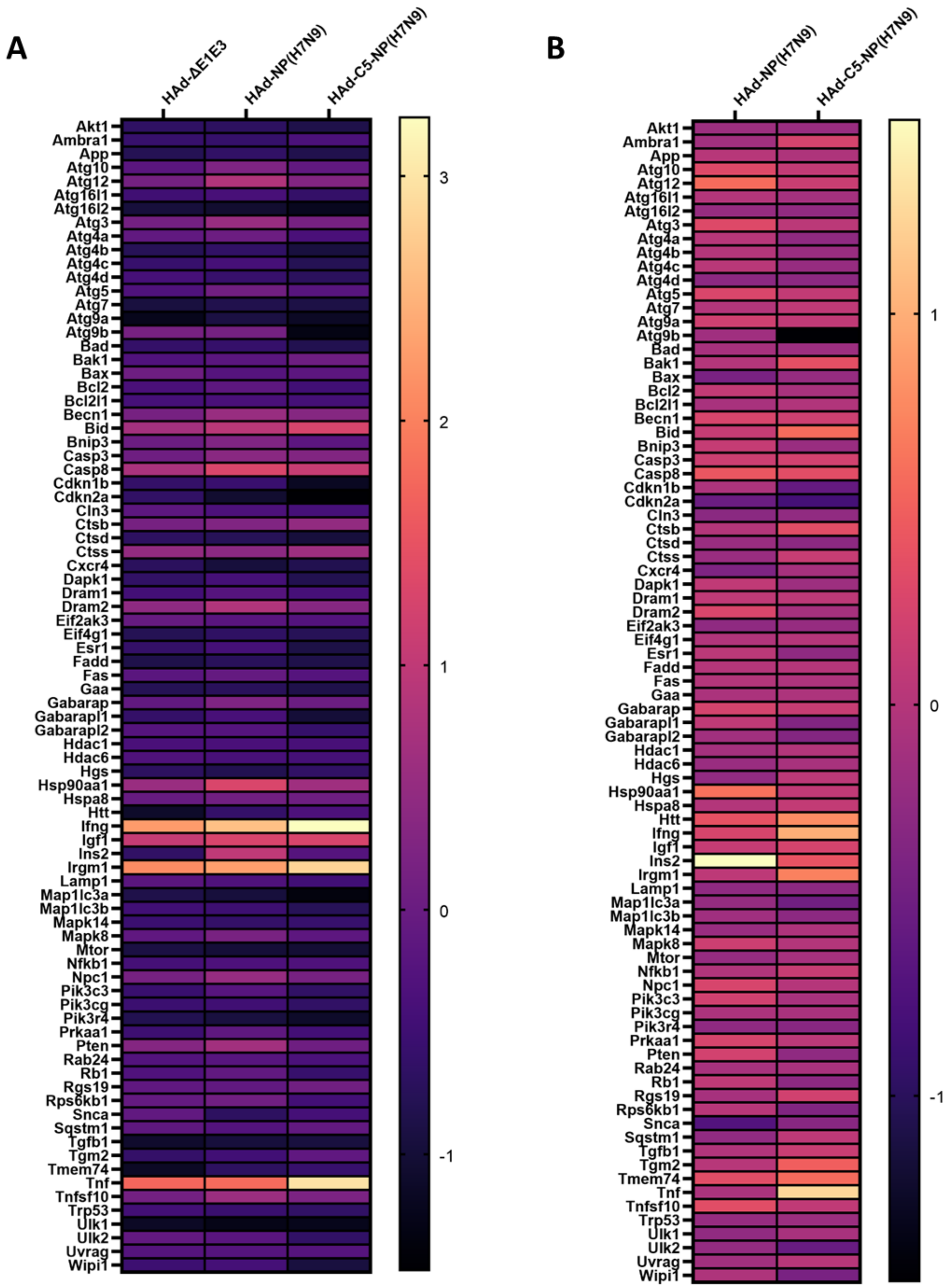


**Figure S1.** Heat maps of the differentially expressed genes with fold change value for HAd-ΔE1E3, HAd-NP(H7N9), or HAd-C5-NP(H7N9) group compared to the PBS group **(A),** and the differentially expressed genes with fold change value for HAd-NP(H7N9), or HAd-C5-NP(H7N9) group compared to the HAd-ΔE1E3 group **(B).**

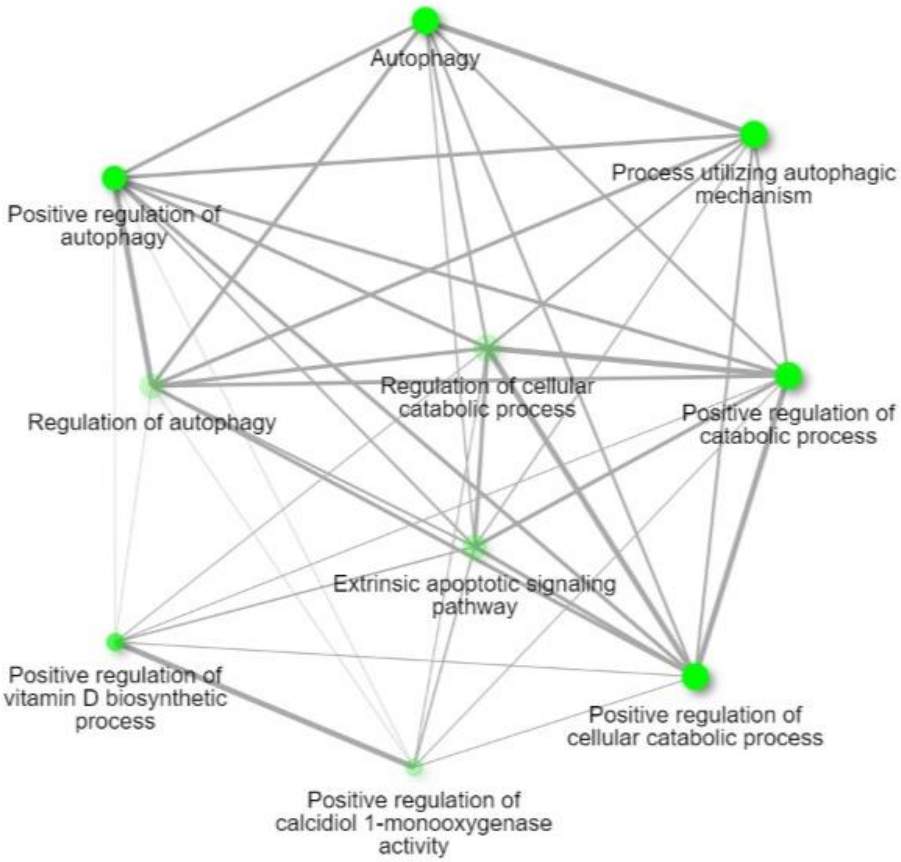


**Figure S2.** Interactive plot showing the relationship between the enriched pathways of the up-regulated genes in the HAd-C5-NP(H7N9) group. Significantly enriched gene sets are displayed with darker nodes. The size of the node represents the relative number of gene sets. Thicker edges represent an increased number of overlapped genes.

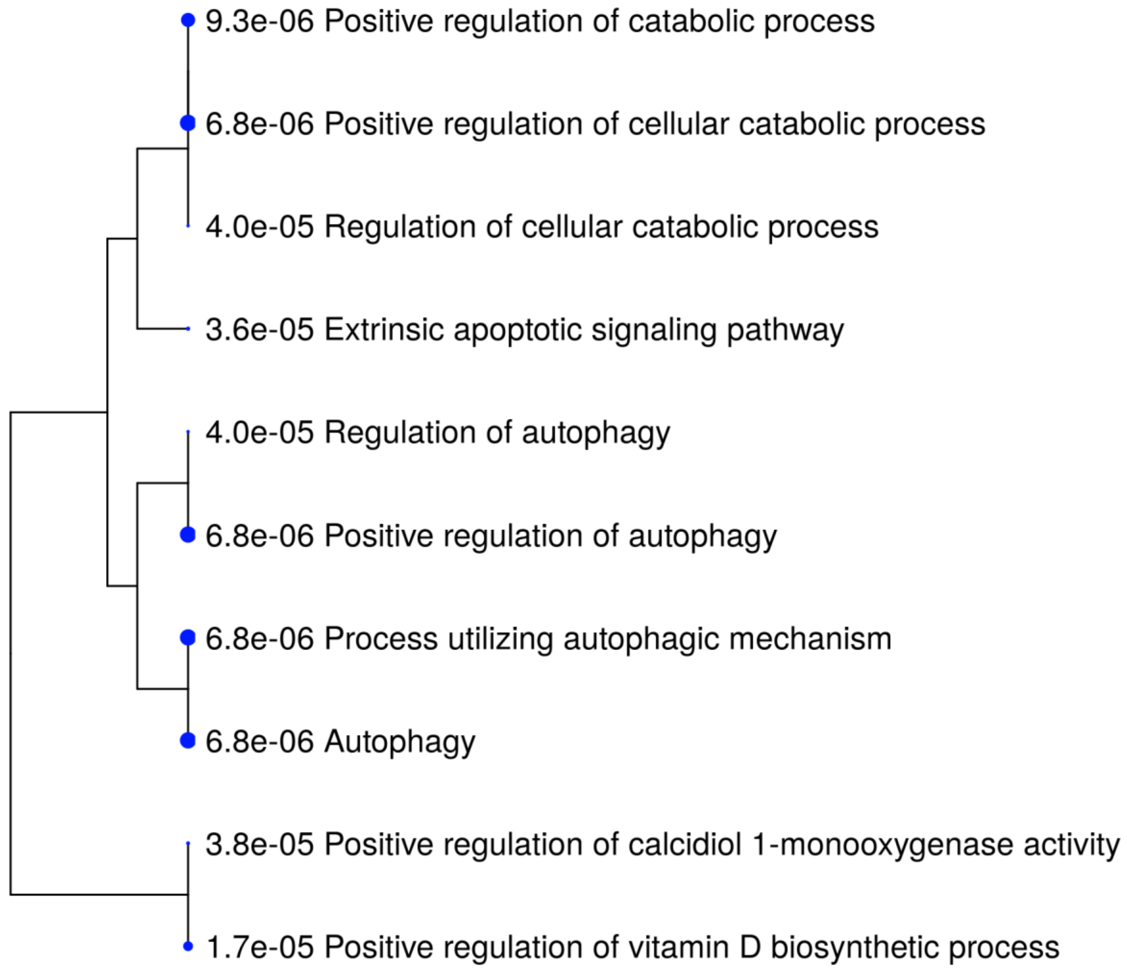


**Figure S3.** A hierarchical clustering tree summarizing the correlation among significant pathways of upregulated genes in the HAd-C5-NP(H7N9) group. Pathways with many shared genes are clustered together. The size of the dots indicates increased *P*-values.
